# Employment Practices, Cost Minimization, and Their Implications for Food Provisions and Seafarers’ Wellbeing on board Ships – A Qualitative Analysis

**DOI:** 10.1177/00469580241229613

**Published:** 2024-01-31

**Authors:** Polina Baum-Talmor, Çağatay Edgücan Şahin

**Affiliations:** 1 Dalhousie University, Halifax, NS, Canada; 2 Ordu University, Ordu, Turkey

**Keywords:** food, seafarers, power dynamics, wellbeing, precariousness, shipping, cost minimization, employment

## Abstract

The global shipping industry, responsible for delivering over 70% of the world’s goods (in volume), has increasingly adopted cost minimization policies, contributing to precarious employment practices that adversely affect seafarers’ wellbeing. This study focuses on the intricate relationship between employment precarity and food provision on cargo ships. By presenting seafarers’ perspectives, we aim to understand how precarious employment practices and cost minimization in the industry influence power dynamics related to food and impact seafarers’ wellbeing. Drawing on empirical data collected through shipboard observations and interviews with seafarers, this study examines the often-overlooked experiences and perspectives of seafarers. The research sheds light on the precarity of employment in shipping and its inherent impact on the provision of food on board and its implications for seafarers’ physical and emotional health, including the availability of nutritious and sufficient food and its impact on their daily lives. Through in-depth interviews, seafarers’ insights into their experiences of food including the quality, availability, and cultural appropriateness of food on board are explored, as well as the standard of training for cooks. Through this research, we found substandard conditions on some of the ships, cost-focused decision-making, and lack of standardized food preparation practices on board. These findings underline the need for improved regulations, better training opportunities, and increased consideration for seafarers’ wellbeing. These changes are essential to ensure the provision of adequate and nutritious meals that promote the physical and mental health of seafarers on board ships. Specifically, the research underscores the need for policy and advocacy initiatives to improve seafarers’ lives and promote fair working conditions in the global shipping industry. By amplifying the voices of seafarers and providing evidence-based insights, this study contributes to the larger discourse on workers’ rights and the importance of decent working conditions. It calls for greater attention to the provision of adequate, nutritious, and culturally appropriate food on board cargo ships, recognizing its significance for seafarers’ physical and mental wellbeing, as well as a call for standardized training for ship’s cooks.

**What do we already know about this topic?**
We know that seafarers face precarious and difficult working conditions at sea.
**How does your research contribute to the field?**
Our paper covers a relatively under researched area of nutrition on board cargo ships and ways in which cost minimization policies and globalization impacted food on board ships, compromising food quality, quantity and cultural appropriateness.
**What are your research’s implications toward theory, practice, or policy?**
Through empirical investigation and data, we provide evidence that shows how the sustenance of seafarers on board standard and substandard ships can be improved, offering practical recommendations toward theory, practice and policy.

## Introduction

The global shipping industry plays a crucial role in delivering over 70% (in volume) of the world’s food and necessities.^1^ It has been previously noted how precarious employment practices are commonly used in this industry,^2-5^ often negatively impacting seafarers’ work conditions and their physical and emotional wellbeing. One important aspect of seafarers’ work relates to food. Seafarers working on board cargo ships often face long voyages which result in limited access to fresh food, and limited control over their diets.^6-11^ Limited access to nutritious food can lead to a range of health problems and a negative impact on seafarers’ overall wellbeing.^9,12,13^ The importance of food has been formally acknowledged in the most recent amendments to the Maritime Labor Convention (MLC) which focused on decent labor and the recognition of the importance of food to seafarer’s wellbeing.^14^

The aim of this paper is to shed light on the current situation regarding food on board cargo ships through the presentation and analysis of empirical data to bring forth the experiences and perspectives of seafarers themselves. By doing so, we hope to gain a better understanding of the ways in which precarious employment in the global shipping industry influences food related power dynamics and seafarers’ wellbeing on board ships. We hope to inform policy and advocacy efforts aimed at improving the lives of seafarers worldwide and ensuring seafarers have access to adequate, nutritious and culturally appropriate food while at sea. Ultimately, we hope to contribute to the larger conversation about workers’ rights and the need for just working conditions in the global shipping industry.

## Cost Minimization and Seafarers

### Global Developments in the Shipping Industry

Sea transport is the dominant method for global merchandise trade,^2,5,15,16^ involving over 90 000 commercial vessels worldwide,^1^ led by over 1.9 million seafarers.^17^ While working conditions for seafarers were relatively stable in the first half of the 20th century,^2,18-21^ employment practices and seafarers’ working conditions began to change in the latter half of the 20th century.^2,5^

Cost minimization policies have shaped the development of the world economy over the last 50 years.^22-24^ These policies are based on supply-side economics which promote export-oriented economies, and financialization which aim to accelerate and intensify capital accumulation by the liberalization of trade and the reduction of local/national market protectionism practices, including labor rights. Accelerating the realization of invested capital is essential for ensuring a consistent supply of goods, making it vital for the extracted raw materials and manufactured goods to be delivered to the buyers as quickly as possible and at the lowest cost.^25-27^ This is where the global shipping industry plays a vital part, accounting for over 70% of the world’s goods (by volume).^1^

There are 5 strategies that characterize the competition for profit of shipping in the late twentieth and early 21st-centuries,^28^ which represent minimization of costs and valorization of capital in shipping. These include investment in large ships that require less crew per ton of cargo, state subsidies, corporate concentration and centralization, flagging out to increase after-tax profits and reduce the cost of complying with labor and other laws, and the precarious employment of seafarers from low-income countries.^28^

The practice of “flagging out” or FOCs (Flags of Convenience) became increasingly common in the 1970s and 1980s, following bankruptcies, mergers and falling freight rates^2,5^ as one way of surviving in a highly competitive global market. Ship registration with FOCs has contributed to a largely precarious work environment for seafarers with minimal regulation of work conditions. This, in turn, impacted seafarers’ general well-being on board,^3,5^ which can also be linked to the structural determinant of food on board.

### Precariousness and food

The Maritime Labor Convention (MLC) addresses global maritime labor regulations, dealing with various issues relating to seafarers’ welfare, from the structure of employment contracts, to the standards of accommodation and food.^29^ While the MLC regulates certain standards of working conditions, it is often challenging to enforce such regulations where shipping companies are faced with a competitive market, looking to maximize profits.^30^ The MLC addresses food and drink however, as Oldenburg et al^9(p192)^ state: “*A critical appraisal of the MLC [. . .] reveals that the nutritional situation on board is neither standardized nor mandatory [. . .], but adapted to the standard of each member state*.”

There are often items of expenditure on shipping vessels that enable companies to save costs while other items make it difficult to do so. For example, paying for provisions and wages is priced regionally by shipping companies and ship owners,^31^ which makes cost minimization and cuts possible.^32^ However, fuel and freight rates are volatile, dependent on the global economic environment,^33-35^ making it harder to control hence to minimize costs. Cost minimization in this respect impacts seafarers’ welfare,^14,29^ impacting the quality and quantity of food on board.

Compared to individuals ashore who can choose their foods from a variety of sources like shops or restaurants, seafarers’ unique and isolated work environment^2,5,36^ means that the food choices offered to them are restricted to what can be found on the ship.^9,11^ Seafarers are likely to eat less “healthy” foods when compared to their food habits ashore,^6-9,11,37^ which can be associated with the stressful work environment experienced by them,^9,38^ or by virtue of the food available to them on board during different phases of their voyage, that is, less fresh fruit and vegetables available after weeks at sea. Despite a growing interest and focus on research related to seafarers,^3,39^ research focusing specifically on the food situation on board ships is limited. To assess the current situation relating to food on board, we analyzed data from interviews conducted with seafarers.

## Methods

The information used in this paper is based on data collected in 3 separate research projects conducted between 2010 and 2023 where supplementary data on food arose during data collection. The focus of the original projects was on seafarers’ welfare and career development, on the determinants of marine officers’ psychology, and on the living and working conditions on board bulk-carriers.

A qualitative approach was used in these projects, including observation on board ships, interviews and conversations with a total of 159 participants. In this paper, we highlight the experiences of 15 participants who characterize the views of the majority of the participants. The choice of the qualitative interpretive approach was chosen as the most appropriate method to yield the data we were seeking. This was also based on previous research projects that studied the global shipping industry,^40-42^ where interviews were used as the main data collection methods and researchers also spent time on board ships to increase trust-based relationships and rapport, often leading to improved quality and richness of interviews.^4^ Participant and non-participant observation was conducted by both authors on board a total of 4 ships. The lead author, who identifies as a woman, sailed on board 3 different ships that were considered standard cargo ships, while the secondary author, who identifies as a man, sailed twice on board one ship that can be considered substandard. Time spent on board ships by researchers ranged between 3 weeks and 3 months. In-depth semi-structured recorded interviews as well as informal non-recorded conversations were also conducted to collect data. Data was analyzed using the Nvivo software, following the code and retrieve method, which involved methodical coding through the raw data.^43^

Both research projects adhered to strict ethical procedures as part of the research institutions in Israel, the UK, and Turkey. All participants signed consent forms after reading information about the respective research projects. All seafarers except a third officer who took part in the projects are male. All data used in the paper is anonymized and pseudonyms are used for all participants. A detailed demographic breakdown about participants can be seen in Tables 1 to 3.

**Table 1.** Distribution of Roles on Board.

Roles on board	Number of seafarers	Percentage of participants (%)
Officers in the deck department	39	24
Non-officers in the deck department	27	17
Officers in the engine department	39	24
Non-officers in the engine department	19	12
Galley/housekeeping department	13	9
Other departments/ roles	22	14
Total	159	100

**Table 2.** Age Groups.

Age groups	Number of seafarers	Percentage of participants (%)
20-29	41	27
30-39	38	24
40-49	29	19
50-59	11	7
60-69	4	3
Ages not disclosed/shared with researcher	36	20
Total	159	100

**Table 3.** Participants’ Nationalities.

Geographical origin	Number of seafarers	Percentage of participants (%)
Azerbaijan	7	4
Bangladesh	1	1
Bulgaria	2	1
China	2	1
Croatia	1	1
Denmark	2	1
Georgia	14	8
India	18	11
Israel	22	14
Iran	1	1
Moldova	1	1
Myanmar	2	1
Poland	1	1
Romania	8	5
Russia	6	4
Sweden	1	1
The Philippines	25	16
Türkiye	30	19
Syria	2	1
United Kingdom	1	1
Ukraine	12	7
Total	159	100

## Results

### Precarious Employment and Its Impact on Food

Precarious work spans formal and informal economies, featuring varying job insecurity levels, including legal status and emotional factors. Seafarers are employed on a contractual basis, facing ongoing job insecurity, reduced pay, and inconsistent benefits tied to employment duration.^5,36^

Regulatory authority at sea is typically influenced by the flag state, with a stronger impact on Scandinavian-flagged vessels navigating between developed maritime nations, while holding minimal significance for ships registered under open registries primarily for profit.^15,28(p113)^ In addition to the seafaring occupation being a precarious one, open flag registries impact job protection on board. For instance, Yakup, an experienced cook who previously worked on an oil drilling tanker (not FoC) referred to the hierarchy on board an open registered vessel, emphasizing the inherent tensions and power dynamics between higher ranked officers and the galley staff on board:

*It’s important that you have to satisfy the Master, the Chief Officer, and the Chief Engineer by your meals. The others are not so important. These three are strong enough to sign you off in the slightest debate or discontent. (Yakup, 46 y.o., 25 years Chief Cook, Türkiye).*

Precarity denotes social and economic insecurity tied to post-Fordist employment and neoliberal governance, allowing employer freedom in hiring and promoting contract-regulation-free part-time, contingent work.^44(pp34-35)^ Even though there is no employer on board, the hierarchical structure of work on board means that there are inherent power dynamics between the officer and non-officer ranks on board.^5,36^ These power dynamics often mean that in addition to wielding significant authority, the captain and other high-ranking officers were also the ultimate decision-makers when it came to retaining crew members. The precarious nature of work on board as noted earlier, combined with these dynamics, have put increased pressure on Yakup to cook good meals, otherwise risking the termination of his contract. However, the higher quality of food mentioned by Yakup was targeted at the officers and not the other seafarers who are “not so important,” creating unequal access to food on board among different ranks.

In this case, the power dynamics between the senior officers and Yakup were evident in day-to-day operations, but Yakup told us how he used his position to shift the power dynamics as a form of resistance:

*I faced the Chief Engineer’s teasing and taunting for three months. [. . .] After three months in 2020, I said to him “I [will] put something in the food, and everyone gets diarrhea; so, you can’t sign off from the ship.” [. . .] He didn’t bother me anymore. (Yakup, 46 y.o., 25 years Chief Cook, Türkiye).*

Yakup’s veiled threat that could potentially lead to a medical emergency is indicative of the unequal measures required to “even out” the power inequality between crewmembers. The threat to temper with the food as a form of resistance links to seafarers’ rights to equal access to safe and hygienic food as covered under the MLC, in particular appropriate health and safety measures to make sure food is hygienic. The practice of checking temperatures on one of the studied ships was routinely falsified, where the recording of the temperatures occurred on paper without physically checking the fridge and freezer, not complying with standards. Şeref shared:

*I don’t ask C/Os (Chief Officers) what to cook on a monthly or even weekly basis. Because I am the one who knows which provisions are going to ruin if they are not cooked in a day or two. I can’t count on the cold storages. I mean, they are working but the thermometers are not working properly. [. . .] we are good on paper. . . (Şeref, 52 y.o., 33 years Chief Cook, Türkiye)*

According to section 3.2 in the MLC^14^ “*Frequent inspections have to be carried out and recorded*” on board to “*ensure that seafarers have access to good quality food and drinking water provided under regulated hygienic conditions*.” The temperature checking was not a reliable practice on the reported ship, in violation of hygiene codes and potentially a risk to seafarers’ health.

This practice could also be linked to the precarious nature of work on board.^3^ Some cooks shared that if they showed the company, they could work with what they have and not complain about the substandard conditions on board, then they were more likely to keep their jobs with the company. For example, Bartu shared:

*Because it’s an old ship, galley hot plates heat up very late, so I have to wake up earlier. Also, the drains, which are very important for cleaning, must be working well. If they’re not, it’s a lot of additional work for the cook and the steward. (Bartu, 26 y.o., 5 years Chief Cook, Türkiye)*

The additional work Bartu had to undertake to compensate for the old equipment on board, was added to his daily workload, which is reportedly already high and intense when working on board cargo ships for extended periods.^3^

### Cost minimization considerations in relation to food

Food provisions should be done in accordance with the MLC regulations, however, in reality, shore-based operations departments often contract food provisioning to one or more specialist ship supply companies while others allow self-purchase, where captains manage the budgets directly.^45^

In their position as the intermediaries between the company and the seafarers on board, captains are often expected to save money for the company while maintaining food and nutrition standards for the seafarers. Cost minimization and regulatory compliance often clash, especially when the nature of agreements between provision companies and shipping companies’ managers is based on minimizing costs, and not on the needs of seafarers on board. Data collected on board substandard ships shows how some ship company managers signed agreements with provision companies ashore to continue to buy supplies from the same provider located in the same geographical location as the company’s headquarters. In return, the shipping company negotiates discounts on provisions in a relationship that can be defined as mutually beneficial.

Seafarers’ welfare is not considered when making these arrangements however and has a substantial impact on food on board. For example, it has been shared by the participants how their food provision company changed some of the items with others within the margins of their agreement, where they sent boxes of turnip juice instead of fizzy sugary drinks, added several low turnover items for the company such as halva (a sesame-based snack) as a way to unload stock, and added additional boxes of ketchup and mayonnaise very close to their expiry date.

The multicultural nature of the crew and different dietary preferences meant that these foods were not readily consumed by the seafarers. In a sense, the provider company attempted to achieve financial gain within the limits of the agreement, which had implications for the cooks and stewards who faced additional pressure daily from seafarers related to food quality and quantity. This also led to food waste where supplies were thrown away almost in every batch, which contradicts section 4.2.1 / 57 in the “Guidelines on the training of ships” cooks’^46^ where it is stated that cooks should “be able to estimate the amount of leftovers and include their use in menus, reducing food wastage both in the longer term and in day-to-day planning.” In this case, even when cooks were trying to implement their training, the circumstances in reality led to reduced quality of food served to seafarers, food waste, and falsification of documents.

Another example where ship operations took priority over seafarers’ welfare was evident in a conversation with a Second Engineer from Turkey:

*“The company told us to get the ship to Turkey safely and not to worry about the rest. The food was already disastrous, I lost 10 kilograms. But everyone, the captain and the Chief Engineer, had the same food, and who would have a reaction to that? [note - meaning to say that if the senior officers did not complain, who am I to complain]” (Bekir, 25 y.o, 3rd Engineer, Türkiye).*

Bekir shared his experience of poor nutrition on board. Other seafarers shared how low-quality food was brought to the ship to cut costs. Zurab shared how one captain compromised seafarers’ wellbeing to maintain a good relationship with the company:

*The captain reduced the ship’s food requirements for the next port. The quality of the food then deteriorated and he left us in a very difficult situation for weeks. He had his own provisions in his cabin from the first day. His aim was to maintain good relations with the company by reducing the ship’s provisions and minimizing costs, so that he could sign another contract there while we suffered. . . (Zurab, 28 y.o., 2nd Officer, Georgia).*

These cases were not uncommon and occurred in other contexts as well. For example, not having enough food was a normal occurrence as described by the cook:

*Deck officers cut the list by saying this is too much and this is unnecessary. Then, if I need 40 kg of rice, I demand 50-60 kg. Because it will be cut. . . Each ship has a provision demand limit that varies according to how many people work and the company does not exceed it. We do it but normally there is no dinner on these ships; you eat your lunch in the evening. (Şeref, 52 y.o., 33 years Chief Cook, Türkiye).*

It was a common practice for the company to cut the suggested provisions in half, a practice that was known by the captain, who doubled the amount of the requested provisions to make sure seafarers had enough provisions on board. In other cases, it was reported how staff on shore took food from the ship’s pantry for their own benefit, often leaving seafarers without sufficient provisions.

### Onboard culinary realities

Opinions on what counts as good or tasty food vary between people, as they would in any other setting. However, the uniqueness of food on board the ship impacts the availability of alternative food options for seafarers at sea. For example, if the food options served at work are limited, people normally have the option to go out and buy a meal somewhere. For seafarers, food options are limited to the ship, as they cannot order food from a take-away restaurant or go out to buy groceries. Food is often perceived as one of the only comforts on board, considering the oftentimes stressful work environment.^3^ Good food, however, is not always considered healthy food as illustrated by Oberyn, a 40-year-old Chief Officer from Ukraine:

*It’s good to have a nice meal, I mean, no one is on a diet here, as a rule I gain 10 kg for every voyage [. . .] Because you don’t have any other enjoyment [. . .] so one of the pleasures [on board] is consuming food. [. . .] I’m busy with the computer, there are constantly decisions to be made, what comes in, what comes out [. . .] so I have to eat properly. That’s why I gain weight, because the food is not always healthy, it has more calories than at home. (Oberyn, 40 y.o. Chief Officer, Ukraine)*

In this respect, in their paper on the changes in seafarers’ health between 2011 and 2016 Sampson et al state how: “. . .*consumption of fried food at sea is far higher than consumption at home*.”^10^ The cook plays an important role in this:

*Everyone is connected in the ship, but the cook is connected to everyone. If I don’t get up one day, for example, the whole crew of 15-20 people will go hungry. So, the cook is actually the most important person on the ship. As there is no TV, internet, phone or anything else aboard, the food is the most satisfying part (Bartu, 26 y.o., 5 years Chief Cook, Türkiye).*

For many seafarers, food is different from what they are used to at home. Lancel, for example, a 24-year-old Electrician from Ukraine shared:

*I’m used to a different diet. [. . .] in Ukraine, in Europe in general. [. . .] I’m used to a certain type of food, like borsht [beetroot soup], pelmeni [dumplings], and it’s not that the food is not fresh, but it’s food I’m not used to. On the previous ship, me and a couple of people bought some buckwheat, and we were just cooking for ourselves, not even going to the mess room. (Lancel, 24 y.o., Electrician, Ukraine)*

Goffman referred to some ships as having similar characteristics to those of “total institutions,” where there is generally “*a breakdown of the barriers ordinarily separating these 3 spheres of life*.”^47^ On board the cargo ship, seafarers generally work, sleep and spend their free time on board, where needs like food, sleep and shelter are supposed to be provided. For Lancel, instead of spending his limited free time resting and unwinding, he spent it worrying about food.

Globalization and cost minimization practices which aim to decrease labor costs as previously mentioned^28,48-50^ are linked to the increased use of multinational crews. For example, there were 25 seafarers on one of the researched ships, with 9 different nationalities. This multicultural crew composition has an immense impact on ways seafarers experience food on board, both from the receiving end as described by Lancel and that of the ship’s cook. One Chief Cook told us about his view of cooking for multinational crews, where he emphasized several times how people needed to be happy with the food on the ship. Stannis, a 50-years-old Chief Cook from India said:

*Every 15 days I prepare food for each country. [. . .] One day I prepare some Chinese style, Chinese noodles, Chinese fried rice, and other day I prepare some Indian, but not too hot, I make it [less] hot, so everybody like [it]. [. . .] I like to make chicken, because chicken I make five or six varieties. (Stannis, 50 y.o., Chief Cook, India)*

The ship’s cook plays a pivotal role in its culinary operations, trained like other seafarers as required by the STCW,^51^ and also undergoes cook-specific basic training.^46^ The precarious nature of work on board^3,36^ and the lack of standardized menus for cooks often puts pressure on cooks to “stand out” compared to other cooks as a coping strategy with this precariousness. For example, Amory, a 50-year-old Chief Cook from the Philippines said:

*It’s my personal hobby to look for other new things, relative to my job. Because in my job this is also a silent competition with other cooks [. . .] no one talks about it, because there is [. . .] no standard food preparation, every cook has his own standard, [. . .] his own responsibilities. [. . .] I managed to survive [in the shipping industry] and so far, that’s the one secret weapon I am using so they [the employer] are keeping me for longer than my colleagues. (Amory, 50 y.o., Chief Cook, The Philippines)*

This is the reality for Amory and other cooks interviewed. The ILO.^46^ guidelines for cooks serve as recommendations for flag state countries, but in practice, as demonstrated in this paper, training is not uniform nor standardized for ship cooks worldwide. As with other training related to seafarers, a cost-cutting practice that has become common among ship owners is reducing investment in vocational training for seafarers,^52^ where seafarers are responsible for their own training. Under these circumstances, cooks often need to fend for themselves when it comes to training, especially if they want to remain competitive in the labor market for cooks at sea, considering the lowering labor demand mentioned and precarious nature of work. On ships that are considered substandard, where culinary needs are not always met as noted previously, cooks need to use their wit and creativity to be able to work with the existing ingredients and serve appropriate food for seafarers:

*Our provisions were generally inadequate; yet, you have to create something. Because of the lack of provisions, I made a pastry with a mixture of creamed potatoes and flour on the inside and flour on the outside. A cook must make such a hustle to survive; without us, people would go hungry. (Cenk, 40 y.o., 15 years Chief Cook, Türkiye).*

Bartering was also described by other seafarers, where the cook on board asked to barter basic supplies with other ships, as described by an engineer:

*Our already limited supplies began to run out. The company told us to wait [on anchorage] until the cargo was loaded, but the remaining time was unpredictable. So, the C/O contacted nearby ships and I, the cook and two sailors visited two other ships to exchange some of the provisions we had for those we didn’t, and vice versa. This was important because the cooks were trying to cook meals that met the daily calorie standards of the seafarers. (Yavuz, 39 y.o., 3rd Engineer, Türkiye).*

In this case, cooks were taking on the responsibility that would normally be that of the shipping company, in an attempt to provide seafarers with sufficient nutrition, in clear violation of section 3.2 of the MLC that refers to sufficient food. These added responsibilities coupled with precarious work conditions were often linked to deterioration in cooks’ quality of work after several weeks at sea. For example, Bekir noted:

*One thing I have seen many times on bulk carriers is that about 10-15 days after joining the ship, the cooks start to sloppily do their job (Bekir, 25 y.o, 3rd Engineer, Türkiye).*

Similarly, another seafarer said:

*[Cooks] have a pattern: they do fancy work in the first week or until the second week. Then you have to deal with their natural way of doing things (Aslan, 31 y.o., Able Man, Azerbaijan).*

Other stories were less anecdotal, where cooks were described as doing unethical things like discarding food that was still usable and in good condition. One chief officer shared:

*I caught the cook [. . .] in the middle of the night on the voyage, throwing a bunch of bananas overboard and I asked him what he was doing, he said that they were rotten. The whole bunch was rotten? Well, then you should have distributed them before they rotted so that people could eat them. [. . .] Throwing away their peels [. . .] was hard for him, that’s what I think. . . (Erkan, 35 y.o., Chief Officer, Türkiye).*

Food waste by cooks on board substandard ships was mentioned by other seafarers, who generally explained it by saying that cooks wanted to lower their workload and not work as hard. Bekir described how one cook:

*Deliberately leaves the door open so that the fruit and vegetables spoil. . . So that he doesn’t have to cut the vegetables [. . .] The company bought three small watermelons for 40 USD each; he cut one and we ate it, but the other two rotted! (Bekir, 25 y.o, 3rd Engineer, Türkiye).*

The cook’s behavior links to food waste, lack of motivation, and precarious working conditions can be explained by previous studies that focused on the expected and perceived workload of workers,^53^ where workers reduced their productivity when they did not feel supported by their employer.

The views from the ship highlight the unique relationship that seafarers have with food while at sea. The limited food options on board, often high in calories and different from what they are accustomed to at home, make food a significant source of comfort in an otherwise challenging work environment. The role of the ship’s cook becomes paramount in providing not only sustenance but also a sense of satisfaction and connection for the multinational crew. However, the lack of standardized training and the pressure to stand out in the highly competitive labor market for shipboard cooks can result in unfortunate consequences such as food waste and declining quality of work. The precarious nature of work at sea, coupled with globalization and cost-cutting practices in the shipping industry, underscores the need for a closer examination of seafarers’ well-being and the role of food in their lives.

## Discussion and Conclusion

Cost minimization perspectives, with their emphasis on market deregulation and individual autonomy, have significantly influenced the global economy and labor market dynamics in the global shipping industry. The precarity of employment that often accompanies cost minimization policies can have detrimental effects on various aspects of seafarers’ lives, including the provision of food on board ships. Cost minimization promotes flexible labor markets, characterized by short-term contracts and outsourcing. While these measures aim to increase efficiency and reduce costs for employers, they often result in heightened job insecurity for workers. Seafarers, who spend extended periods at sea away from their families, are particularly vulnerable to the precariousness of employment fostered by cost minimization practices.

Precarious employment has been shown to directly impact the food situation on board ships. As highlighted in the results section, cooks and other crew members are frequently subjected to unpredictable schedules, extended work hours, and heavy workloads. Goffman referred to some ships as having similar features to those of “total institutions,” where there is “*a breakdown of the barriers ordinarily separating these 3 spheres of life*.”^47^ On board the cargo ship, seafarers generally work, sleep and spend their free time on board, where needs like food, sleep and shelter are supposed to be provided. Often, food is perceived as one of the only comforts on board, considering the oftentimes stressful work environment.^3^ These conditions, coupled with the lack of employment stability, pose challenges for maintaining adequate and nutritious meals on board. Cooks were shown to struggle to allocate sufficient time for meal preparation and have limited access to fresh ingredients, impacting the variety and quality of meals served. When it comes to reports of consuming unhealthy foods on board, it has been stated by Sampson et al in their paper on the changes in seafarers’ health between 2011 and 2016, how: “. . .*consumption of fried food at sea is far higher than consumption at home*.”^10^

Reports on staff on shore who took food from the ship’s pantry was also described in Sampson et al who described how *“port-based personnel had no shame in raiding provisions from the storerooms and no regard for the fact that this could leave the seafarers without goods which the port personnel could readily purchase ashore yet which once removed from the vessel were out of reach of the seafarers*”.^54(p16)^ Not having enough provisions or poor provisions of food on board was also mentioned in the media.^55,56^ These examples address the reported cases, however there are many undocumented cases that do not reach the media nor the authorities. Moreover, the absence of stable employment contracts can result in frequent turnover among cooks, leading to a lack of continuity in culinary skills and knowledge, and potentially compromising the consistency and standard of meals served to seafarers.

In terms of precariousness, the data shown in the results section links to previous reports on a decline in the number of seafarers working in ship galleys, attributed to cost-cutting measures in the shipping industry, resulting in smaller crews.^5^ The financial strain caused by precarious employment restricts shipping companies’ ability to provide sufficient resources for onboard catering. Limited budgets may lead to cost-cutting measures in food provisioning, such as purchasing lower-quality ingredients or reducing the variety of food options. This, in turn, affects the overall satisfaction of meals, contributes to health issues among seafarers, and can be associated with cooks wasting food to avoid workload as documented by seafarers.

Furthermore, the lack of job security and the fear of job loss can create an environment where seafarers, cooks and captains alike, may hesitate to voice their concerns about food quality or raise complaints regarding the provision of meals. This fear of reprisals or non-renewal of contracts may discourage open communication between crew members and management, making it challenging to address and rectify any issues related to onboard food provision.

The purpose of this paper was to shed light on the current situation on board ships in relation to food and make recommendations for stakeholders in the global shipping industry on how to improve the existing situation. The precariousness of employment on board ships significantly influences the food situation and quality experienced by seafarers.

The hierarchical power dynamics on board often dictate the cook’s priorities in satisfying senior officers who hold the authority to determine their future employment. This power dynamic can lead to substandard conditions and compromised food quality. The reported reliance on inadequate temperature checking and malfunctioning equipment in the galley contributes to potential health risks and compromises hygiene standards.

Economic considerations focused on cost minimization rather than on seafarers’ welfare also impact the food situation on board. Agreements between provision companies and shipping companies prioritize cost reduction, leading to limited variety, poor-quality items, and food wastage. The multicultural nature of the crew further confounds the provision of suitable meals, as different dietary preferences and cultural backgrounds must be considered.

From the perspective of the ship’s cook, food holds immense importance as it serves as a source of satisfaction and comfort for the seafarers in a stressful and isolated work environment. The cook’s responsibility extends beyond mere sustenance, with the preparation of diverse meals catering to various nationalities on board. However, standardized menus and recipes are lacking, and cooks must rely on their own training to satisfy the diverse crew’s culinary needs.

Overall, the substandard conditions, cost-focused decision-making, and lack of standardized food preparation practices underline the need for improved regulations, better training opportunities, and increased consideration for seafarers’ wellbeing. These changes are essential to ensure the provision of adequate and nutritious meals that promote the physical and mental health of seafarers on board ships.

Based on the issues highlighted in this paper, we offer several recommendations. Firstly, we would recommend maritime training institutions offer standardized training programs for cooks on board, and for shipping companies to invest in such training, ensuring ship cooks receive proper culinary training across the board, including food safety certification, and knowledge of dietary requirements for diverse crew members. This can improve the overall quality of meals and address specific dietary preferences or restrictions. Secondly, shipping regulatory bodies should consider developing standardized menus and recipes that consider nutritional requirements, cultural diversity, and crew preferences. This can help ensure consistent meal quality, variety, and adherence to relevant dietary guidelines while providing flexibility for cooks while maintaining their creativity and professional development. Thirdly, we would recommend future researchers to develop some of the concepts in this paper in addressing nutritional and culinary aspects of food consumption and energy expenditure among seafarers from a holistic perspective that considers the social, cultural and health aspects of food.

We encourage readers of this paper to continue exploring issues relating to food on board ships, considering the complexities. Further exploration of food on board could include an exploration of seafarers’ meals and calorie consumption, further investigate education relating to cooks’ training on board in a comparative study across several geographical areas, and address food provisioning in different contexts including potential gaps between regulations and implementation.
